# Fe_3_O_4_ Nanoparticles in Targeted Drug/Gene Delivery Systems

**DOI:** 10.3390/ma11020324

**Published:** 2018-02-23

**Authors:** Lazhen Shen, Bei Li, Yongsheng Qiao

**Affiliations:** 1 School of Chemistry and Environmental Engineering, Institute of Applied Chemistry, Shanxi Datong University, Datong 037009, China; 13653621281@163.com; 2 Department of Chemistry, Xinzhou Teachers University, Xinzhou 034000, China

**Keywords:** Fe_3_O_4_ nanoparticles, synthesis, functionalization, coating, drug/gene delivery systems

## Abstract

Fe_3_O_4_ nanoparticles (NPs), the most traditional magnetic nanoparticles, have received a great deal of attention in the biomedical field, especially for targeted drug/gene delivery systems, due to their outstanding magnetism, biocompatibility, lower toxicity, biodegradability, and other features. Naked Fe_3_O_4_ NPs are easy to aggregate and oxidize, and thus are often made with various coatings to realize superior properties for targeted drug/gene delivery. In this review, we first list the three commonly utilized synthesis methods of Fe_3_O_4_ NPs, and their advantages and disadvantages. In the second part, we describe coating materials that exhibit noticeable features that allow functionalization of Fe_3_O_4_ NPs and summarize their methods of drug targeting/gene delivery. Then our efforts will be devoted to the research status and progress of several different functionalized Fe_3_O_4_ NP delivery systems loaded with chemotherapeutic agents, and we present targeted gene transitive carriers in detail. In the following section, we illuminate the most effective treatment systems of the combined drug and gene therapy. Finally, we propose opportunities and challenges of the clinical transformation of Fe_3_O_4_ NPs targeting drug/gene delivery systems.

## 1. Introduction

Targeted drug/gene delivery refers to nanocarriers carrying drugs/genes to organs, tissues, and cells through local or systemic blood circulation, which allows the drugs/genes to directly act on the targeted disease sites, accompanied by the generation of curative effects. This selective administration boosts therapeutic molecule activity at targeted sites, while reducing toxic side effects at non-disease sites, thus keeping the systemic effect at a minimal level. The application of nanotechnology in many medical areas has been widely developed, especially in the field of drug/gene delivery [1,2,3,4,5,6,7,8]. The use of nanoparticles as carrier systems for drugs or other bioactive therapeutic molecules has been investigated with the aim of improving the therapeutic effect and administration of the loaded agents and reducing their side effects. Among these nanoparticles, Fe_3_O_4_ nanoparticles (NPs) are used extensively in various fields, including biotechnology [9], biosensing [10], catalysis [11], magnetic fluids [12], separation techniques [13], energy storage [14], and environmental modification [15]. Applications of Fe_3_O_4_ NPs in the field of biotechnology involve targeted drug/gene delivery [16,17,18,19], magnetic resonance imaging (MRI) [20,21], contrast enhancement and hyperthermia reagents [22], biophotonics [23,24], and detection, diagnosis, and magnetic field–assisted radiation treatment of cancerous cells [25,26]. Targeted drug/gene delivery systems are particularly beneficial due to their unique magnetic properties, extremely low toxicity, excellent biocompatibility, good biodegradability, and reactive surface that can be readily modified with biocompatible coatings. Compared with other magnetic materials, Fe_3_O_4_ NPs are preferred because of the presence of the Fe^2+^ state, which has the potential to act as an electron donor. Furthermore, no hysteresis is produced, so they leave behind zero residual magnetization after an external magnetic field is removed. This property helps to avoid coagulation, which consequently lowers the possibility of agglomeration in vivo [27]. Using an external magnetic field and microwave radiation near the tumor tissue, Fe_3_O_4_ NPs can release drugs/genes and absorb microwave energy, rapidly converting the microwave energy into heat for thermal therapy. The temperature of the tumor increases, which can change the structure of lipids and proteins, improve the permeability of cell membranes, promote the entrance of drugs/genes into tumor cells, and enhance the effects of chemotherapy, hyperthermia, and gene therapy.

However, Fe_3_O_4_ NPs possess high surface energy, leading to aggregation, which minimizes the surface energy. Additionally, naked Fe_3_O_4_ NPs have high chemical activity on their surface but are highly prone to oxidization in air, which can lead to significant reduction of their magnetism and dispersibility. Therefore, it is important to consider the functionalization of Fe_3_O_4_ NPs with diverse thin and thick materials, such as all kinds of polymers [25,28,29,30,31,32], graphene oxide materials [33,34,35], silica [21,36], carbon materials [37,38], metal oxide microwave-absorbing materials [39], and various luminescent materials [21,40]. The modified coatings improve the stability and dispersibility of naked Fe_3_O_4_ NPs and provide chemical coordination/conjugation sites for drugs, targeted ligands, genes, and other therapeutic reagents that can optimize the biomedical utilization of targeted drug/gene delivery.

In this review, we first focus on recent developments in the synthesis of Fe_3_O_4_ NPs and various coatings for protection of the particles against oxidation, and then summarize the applications of such Fe_3_O_4_ NPs in targeted drug or individual gene delivery systems in detail. Finally, we describe targeted drug and gene co-delivery systems to attain the most powerful combined therapy.

## 2. Synthesis of Fe_3_O_4_ NPs

Fe_3_O_4_ NPs can be synthesized by either top-down (mechanical attrition) or bottom-up (chemical synthesis) approaches. The chemical methods, including co-precipitation, thermal decomposition and/or reduction, solvothermal synthesis, and micelle synthesis, are better suited to produce nanoparticles with uniform composition and size, and are summarized in Table 1.

### 2.1. Co-Precipitation

Fe_3_O_4_ NPs were first fabricated by the co-precipitation method researched by Massart [41]. Co-precipitation is the most common preparation method to fabricate Fe_3_O_4_ NPs from aqueous Fe^2+^/Fe^3+^ salt solutions with a base at room temperature or at elevated temperature. Here, Fe_3_O_4_ NPs are formed by a nucleation and growth mechanism. At present, Fe_3_O_4_ NPs have been widely synthesized by co-precipitation because of the ease of implementation and the use of less hazardous materials and procedures.

**Figure 1:**
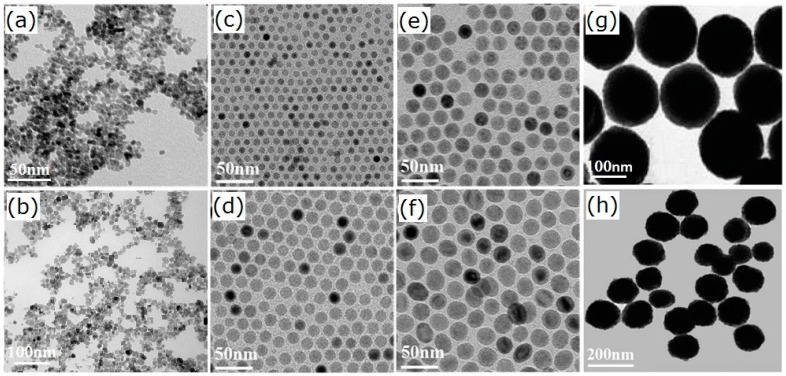
Transmission electron microscope (TEM) images of Fe_3_O_4_ NPs prepared by (**a,b**) co-precipitation method [23,44]; (**c**–**f**) thermal decomposition method [50]; and solvothermal method (**g**) as in [54], (**h**) as in [39].

Liu et al. prepared Fe_3_O_4_ NPs by co-precipitation: a clear solution of FeSO_4_·H_2_O and Fe_2_ (SO_4_)_3_ with a molar ratio of 2:1 was heated to 50 °C with the protection of N_2_, followed by the addition of ammonia aqueous solution under vigorous stirring [42]. Rezayan et al. prepared Fe_3_O_4_ NPs by the same method and verified the ultrasonic clue effect [43]. They showed that homogeneous particles and narrow particle size distribution can be obtained by the ultrasonic technique. According to the particle size distribution curve, the average diameter of nanoparticles was 24 nm in the absence of ultrasonic waves, while the average particle size in the presence of ultrasonic waves was 16 nm, which confirmed that ultrasonic has good efficiency. More uniform Fe_3_O_4_ NPs, with a mean particle size of 12 nm, were synthesized by Voronin et al. according to Massart, as shown in Figure 1a [23]. During the synthesis, an inert atmosphere was preserved continuously, and upon preparation, the particles were stabilized with 0.1 M citric acid and afterward dialyzed in water for 4 days. However, the above co-precipitation methods are inseparable from the inert atmosphere such as nitrogen, and the preparation process is complicated. Our group adopted a simple co-precipitation method to synthesize Fe_3_O_4_ NPs with an average particle size of 10 nm and narrow size distribution (Figure 1b) [44]. First, an aqueous solution of Fe^2+^/Fe^3+^ was prepared by dissolving 3.6 g of anhydrous Fe_2_(SO_4_)_3_ and 3.3 g of FeSO_4_·7H_2_O in 100 mL distilled water to be a homogeneous dark orange solution with magnetic stirring. An aqueous NaOH solution (5.1 g NaOH dissolved in 50 mL distilled water) was then injected into the above solution under vigorous stirring. The reaction was allowed to proceed with stirring for 5 h at room temperature. The obtained Fe_3_O_4_ NPs monodispersed well and were nearly uniform in dimension.

### 2.2. Thermal Decomposition

Using the metastability of compounds, monodispersed Fe_3_O_4_ NPs of smaller size can be fabricated through the thermal decomposition of organic iron compounds in high-boiling organic solvents containing surfactants. The ordinary iron precursors include iron acetylacetonate (Fe(acac)_3_) [19,45], iron oleate [46,47], and carbonyl iron [11].

Kim et al. used an iron oleate precursor and oleic acid as a stabilizing agent to obtain Fe_3_O_4_ NPs stabilized with oleic acid [48]. However, Fe_3_O_4_ NPs prepared by thermal decomposition are often water-insoluble. Zhang et al. synthesized water-soluble Fe_3_O_4_ NPs by thermal decomposition of Fe(acac)_3_ in polyethyline glycol (PEG) in the presence of polyethylenimine (PEI) [49]. Coated with PEG and PEI, the Fe_3_O_4_ NPs were positively charged and highly soluble in deionized water, with excellent colloidal stability. The diameter range of Fe_3_O_4_ NPs also could be tuned from 9 nm to 14 nm by changing the amount of PEI, reaction temperature, and time. The specifically monodispersed Fe_3_O_4_ NPs were fabricated from thermal decomposition of iron carboxylate salts in a mixture of oleic acid and 1-octadecene at 320 °C. The size of Fe_3_O_4_ NPs can be precisely controlled by changing the concentration of initial iron precursor and oleic acid. When FeO(OH) was increased from 2 to 10 mmol, the particle size increased from 8 to 25 nm, as shown in Figure 1a–d [50]. Although thermal decomposition can fabricate high-quality Fe_3_O_4_ NPs, there are also some limitations to this method. It needs relatively expensive organic iron compounds as precursors, and the reaction process needs a high temperature and is a tedious procedure, which hinders large-scale production and application.

### 2.3. Solvothermal Synthesis

The hydrothermal method is the most popular wet chemical approach to synthesizing inorganic nanocrystals from aqueous solution under relatively high temperature and high pressure in a sealed pressure vessel. Using organic solvent to replace aqueous solution under similar conditions to fabricate nanoparticles is called the solvothermal method. In general, the method employs an iron salt, reducing agent/oxidizing agent, acid salt, and one or more surfactants in a reaction system under high temperature to prepare Fe_3_O_4_ NPs [51,52,53].

Deng et al. first reported the synthesis of single-crystalline magnetic Fe_3_O_4_ NP microspheres by a solvothermal reduction method, as shown in Figure 1g [54]. The as-obtained Fe_3_O_4_ NPs also monodisperse and are hydrophilic, and the particle size can be tunable in the range of 200–800 nm with increased reaction time. Li et al. reported the synthesis of Fe_3_O_4_ NPs by the solvothermal method [55]. Typically, a certain amount of FeCl_3_·6H_2_O and NaAc were dissolved in ethylene glycol (EG) solution. Then PEG-10000 was added with vigorous stirring, and the mixture was stirred to form a homogeneous russet solution. The obtained solution was transferred to a Teflon-lined stainless steel autoclave and heated at 200 °C for 10 h. The prepared Fe_3_O_4_ NPs had a rough appearance and uniform size distribution, with an average size of 290 ± 20 nm, and had good dispersity. Then functionalization of Fe_3_O_4_ NPs was done with silica and lanthanide-doped nanomaterials, which endow them with great potential for application in drug delivery, MRI, and marking and separating of cells in vitro. Gao et al. reported a simple one-pot hydrothermal method for the synthesis of carboxyl-modified Fe_3_O_4_ NPs (Fe_3_O_4_@COOH) [56]. Qiu et al. prepared magnetic Fe_3_O_4_ NPs with a mean particle size of 170 nm using the solvothermal method (Figure 1h) [39]. These Fe_3_O_4_ NPs were coated with ZnO and mesoporous silica in turn to obtain a novel core-shell structured Fe_3_O_4_@ZnO@mSiO_2_ nanocarrier, used as a drug carrier to investigate the storage and controllable release properties of the chemotherapeutic cancer drug etoposide.

The size of nanoparticles must be considered as a factor parameter when Fe_3_O_4_ nanoparticles are used for targeted drug/gene delivery systems. The size of these nanoparticles should be large enough (>10 nm) to avoid renal filtration and rapid permeability. Meanwhile, to eliminate the reticular endothelial system phagocytosis of spleen and liver, the diameter of these nanoparticles must be less than 200 nm. Thus, 10–200 nm is a suitable size range for targeted drug/gene delivery systems; nanoparticles in this size range can circulate steadily in blood and further accumulate in tumor sites, and then the target effects are realized and the blood circulation time of the drug/gene is prolonged. Typically, the size of Fe_3_O_4_ nanoparticles is related to the synthesis method. Compared to the co-precipitation method, the solvothermal method can yield more uniformity, with superior dispersibility and magnetic properties. Nevertheless, Fe_3_O_4_ NPs prepared by the solvothermal method have a large mean particle diameter, which blocks their application in targeted drug/gene delivery systems. The size of Fe_3_O_4_ NPs prepared from co-precipitation and thermal decomposition is suitable for drug/gene delivery systems, but thermal decomposition requires hazardous materials and procedures, so the co-precipitation method continues to be the most widely used for drug/gene delivery systems, because the reagents of this method are less dangerous and inexpensive, and the operation is handy and easy to implement.

## 3. Functionalization of Fe_3_O_4_ NPs

Owing to the high surface area and dipole–dipole interaction, Fe_3_O_4_ NPs tend to agglomerate, their particle size increases, and uptake by the reticuloendothelial system (RES) increases in vivo. In order to overcome these shortcomings, it is necessary to explore a suitable method for the functionalization of Fe_3_O_4_ NPs. To confer intriguing physicochemical properties on functionalized Fe_3_O_4_ NPs, the surface of the nanoparticles has been coated by diverse polymers [29,31,57], inorganic materials [58,59], and/or biological molecules [60,61]. Various modified Fe_3_O_4_ NPs for targeted drug delivery under different responsive conditions are listed in Table 1.

### 3.1. Polymer Coating

Modification of the surface of Fe_3_O_4_ NPs with hydrophilic natural polymers such as chitosan (CS), starch, or dextran, or synthetic polymers such as polyethylene glycol, polyethylene phthalate, polyvinyl pyrrolidone, or other distinctive polymers was done to reduce the oxidation, agglomeration, and toxicity and provide multifunction treatments [28,32]. These kinds of composites can effectively deliver therapeutic molecules to target sites and then achieve targeted drug/gene delivery.

Chitosan is a cationic polysaccharide sourced from partial deacetylation of chitin, which is a co-polymer consisting of 2-amino-2-deoxy-d-glucose and 2-acet-amido-2-deoxy-d-glucose units with β-(1-4) linkages. Functionalized Fe_3_O_4_ NPs with chitosan will possess better biocompatibility and low toxicity, and will be able to interact with biological molecules such as polypeptides, DNA, and antibodies. Thanks to its special properties like long polymeric chains, dissolvability in water, and nontoxicity in blood, polyethylene glycol (PEG) is widely used in the field of medicine. Polyvinylpyrrolidone (PVP), a water-soluble polymer with amphiphilic features, also has the characteristics of toxicological harmlessness, film formation, and solubility and can be used as a binder. It can be employed in pharmaceutical, medicine, and industrial production. Prabha et al. synthesized Fe_3_O_4_ NPs by co-precipitation, and then CS, PEG, and PVP were successively modified to form Fe_3_O_4_–CS, Fe_3_O_4_–CS–PEG, and Fe_3_O_4_–CS–PEG–PVP nanocomposites, respectively [67]. The anticancer drug curcumin (Cur) was loaded with these nanocomposites. Under the same initial concentration of curcumin, the electrostatic attraction between nanocomposites and curcumin increased with an increased number of coatings. Later, the encapsulation efficiency (EE) and loading capacity (LC) of Fe_3_O_4_–CS–PEG–PVP nanocomposites were somewhat higher than those of the Fe_3_O_4_–CS and Fe_3_O_4_–CS–PEG nanocomposites. The cancer cells treated with the curcumin-loaded magnetic composite NPs displayed less cytotoxicity when compared to free curcumin, which was attributed to the slower release of curcumin from NPs and a decreased interaction of the drug with the cell wall. Hence the poisonous effects of the drug were reduced. These results suggest that the curcumin loaded on these NPs had the potential for application in cancer therapy. Kievit et al. used a co-polymer of CS, PEG, and PEI to modify Fe_3_O_4_ NPs, and then the as-prepared nanoparticles were labeled with targeted ligand chlorotoxin and conjugated plasmid DNA for targeted gene therapy of glioma. Thus, gene treatment efficiency would be promoted via the use of nanoparticles with an external magnetic field [80].

Starch, a natural water absorption polymer, has the characteristics of biocompatibility, biodegradability, and nontoxicity, and can be used as an effective capping agent. Hamidian et al. synthesized an Fe_3_O_4_/starch-g-polyethylene phthalate hydrogel nanocomposite by graft co-polymerization, and starch was used as a biopolymer backbone [70]. The hydrogel polymeric networks formed could effectively release the drug by gradual hydrolysis. Thus, starch containing Fe_3_O_4_ NPs were grafted with hydrogel polymeric networks to prevent nanoparticle aggregation, and then were loaded with heteropoly acid for drug delivery.

Because the temperature of cancer cells is higher than normal cells, and their pH is more acidic than normal cells (pH 5.8), pH and thermosensitive polymers can be used for selective release of anticancer drug in cancer cells. The drawbacks of this type of polymer are the lack of surface area, which leads to small drug uptake, and the inability to target specific tissue. These problems can be avoided by grafting the polymers on the surface of nanoparticles, especially Fe_3_O_4_ NPs, since they can be delivered to specific tissue under the external magnetic field. Habibi et al. first prepared Fe_3_O_4_ NPs by the co-precipitation method, and 3-(trimethoxysilyl) propyl methacrylate (TMSPMC) was used to prepare functionalized Fe_3_O_4_ NPs (Fe_3_O_4_ NP/TMSPMC) [62]. Then, doxorubicin solution in water was added to the excess amount of pH and thermosensitive polymer *N*-isopropylacrylamide solution in ethanol to synthesize the *N*-isopropylacrylamide/doxorubicin (NIPAAM/DOX) complex. Finally, the poly NIPAAM@Fe_3_O_4_ NP/TMSPMC/DOX was synthesized by radical polymerization of the Fe_3_O_4_ NP/TMSPMC and NIPAAM/DOX complex using azobisisobutyronitrile and ethylene glycol dimethacrylate. The results of drug release studies showed that the amount of released DOX from the polymer increased from 83% to 94% by tuning the pH from 7.4 to 5.8, and the DOX release rate accelerated significantly by tuning the temperature from 37 to 40 °C. As a result, the magnetic nanoparticle polymer composite could be exploited as a controlled drug release carrier for anticancer drugs.

The photothermal performance, colloidal stability, physiological stability, and biocompatibility of Fe_3_O_4_ NPs could be improved by coating with photothermal polymer layers, such as polydopamine (PDA), polypyrrole (Ppy), or polyaniline. Ge et al. fabricated PDA-coated Fe_3_O_4_ NPs (Fe_3_O_4_@PDA), and the photothermal property and superparamagnetism of Fe_3_O_4_@PDA were obviously enhanced due to the collective effect of preassembled Fe_3_O_4_ and PDA [48]. The Fe_3_O_4_@PDA possessed the outstanding property of photothermal ablation of HeLa cells in vitro, and cell viability could be reduced to 35% under laser irradiation. Thus, a new nanodevice for magnetic resonance imaging-guided noninvasive tumor diagnosis and hyperthermic therapy was constructed. Liu et al. employed PDA to encapsulate Fe_3_O_4_ NPs and loaded an absorbed heat shock protein 70 (HSP70) inhibitor to develop a nanoplatform for increased photothermal inactivation of bacteria [29]. The inhibitor could be released from the nanoplatform via the weakening of π-π and hydrogen bonds under near-infrared (NIR) light irradiation, then the function of HSP70 would be limited and the tolerance of bacteria to photothermal therapy was reduced, with an improvement in treatment effect against infectious bacterial pathogens. Thus, the nanoplatform could also bond with other drugs and be used as a recyclable photothermal agent for improved hyperthermic treatment without causing secondary pollution. Ppy not only has an NIR light absorption peak between 700 and 900 nm, it also exhibits excellent biocompatibility, superior conductivity, good stability, and effortless synthesis processes, which could be utilized for photoacoustic imaging and provide a conversion agent for photothermal treatment of cancer. Meanwhile, it can be connected with hydrophobic drugs due to its hydrophobic and π-π stacking properties. However, it has a higher dose of irradiation and limited laser irradiation penetration depth in tissues. To overcome these problems, Tang et al. prepared multifunctional magnetic Fe_3_O_4_@Ppy nanoparticles via an in-suit chemical oxidation polymerization method, and then functionalized them with hyaluronic acid through electrostatic interaction and loaded a Notch signaling pathway inhibitor to eliminate cancer stem cells [31].

### 3.2. Silica/Mesoporous Silica (SiO_2_/mSiO_2_) Coating

**Figure 2:**
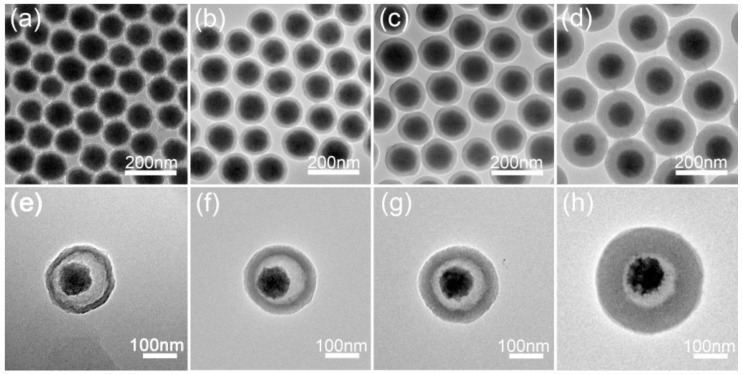
TEM images Fe_2_O_3_@SiO_2_ microspheres with different SiO_2_ shell thicknesses: (**a**) 15 nm; (**b**) 35 nm; (**c**) 50 nm; and (**d**) 80 nm; and (**e**–**h**) their corresponding TEM images of rattle-type Fe_3_O_4_@SiO_2_ hollow microspheres [83].

Due to its nontoxicity, chemical stability, intermolecular redox reactions, and a number of surface hydroxyl groups displaying coordinate sites for further modification [81,82], inorganic silica material is used as a protective and modified shell to coat with Fe_3_O_4_ NPs. Shahabadi et al. synthesized silica-modified Fe_3_O_4_ NPs with the Stöber method via the sol-gel process, on the basis of the hydrolysis and condensation of tetraethyl orthosilicate (TEOS) [68]. The anticancer drug cytarabine was bound on the surface of Fe_3_O_4_@SiO_2_ NPs via hydroxyl groups of the drug, which enhanced the antiproliferative effect of the drug. Peapod-like Fe_3_O_4_@SiO_2_ NPs (p-FS) and traditional spherical Fe_3_O_4_@SiO_2_ NPs (s-FS) obtained from Fe_3_O_4_ NPs of ~80 nm and ~200 nm, respectively, were synthesized by Wang et al. [36]. The p-FS-PGEA (poly(glycidyl methacrylate)) carriers possessed superior gene transfection properties compared with ordinary s-FS-PGEA, which was due to the p-FS nanoparticles exhibiting larger contact area with the cell membrane, so that internalization and cellular uptake were improved. The efficiency of gene transfection could be further facilitated under a magnetic field. Hence the new peapod-like Fe_3_O_4_@SiO_2_ NPs were constructed for effective gene delivery and real-time imaging, and were better than ordinary spherical Fe_3_O_4_@SiO_2_ NPs. Cheng et al. also constructed a new rattle-type Fe_3_O_4_@SiO_2_ hollow microsphere (Figure 2) for targeted drug delivery through template-etching and hydrogen/nitrogen atmosphere-reduction routes [83]. To investigate the feasibility of these microspheres in targeted drug delivery, DOX was chosen as a drug model. With increased void volume or decreased shell thickness, the ability of drug loading increased, and the drug release capability followed the same change rules. It provided not only an easier and more practical route for efficient control of drug release amount and rate, but also a novel synthetic method for the preparation of rattle-type magnetic hollow microspheres with different shell thickness and void volume.

Kim et al. reported the fabrication of silica-coated Fe_3_O_4_ NPs through a reverse microemulsion method, and polyoxyethylene(5)-nonylphenyl ether (Igepal CO-520) was employed as a surfactant and TEOS as a silica precursor [48]. Liu et al. reported the preparation of mSiO_2_-coated Fe_3_O_4_ NPs based on the basic hydrolysis of a silicon source, with TEOS as the silica precursor and *N*-cetyltrimethylammonium bromide as a template [42]. The average diameter of the as-prepared mSiO_2_@Fe_3_O_4_ NPs was 400 nm, and the mSiO_2_ shell was about 300 nm. The resultant nanoparticles were assembled with DNA for monitoring of DNA methyltransferase (Mtase) activity. The nanoparticles could also be employed for other DNA Mtase monitoring, such as cancer-related DNA Mtase, through changes in the corresponding DNA sequence. Thus, the nanoparticles can be used not only to detect DNA Mtase activity, but also for disease diagnosis and drug development.

In conclusion, the obtained new peapod-like Fe_3_O_4_@SiO_2_ NPs, rattle-type Fe_2_O_3_@SiO_2_ hollow microspheres, and mSiO_2_@Fe_3_O_4_ NPs exhibited excellent characteristics, such as superior specific area, more space for drug/gene loading, and outstanding cell internalization, which were preferable over traditional spherical Fe_3_O_4_@SiO_2_ NPs and added a fresh perspective to targeted drug/gene delivery systems.

### 3.3. Graphene Oxide Coating

In all kinds of graphene-based materials, a single layer of carbon atoms are tightly packed in a two-dimensional honeycomb crystal lattice structure. The applications of graphene-based materials have developed quickly, including drug/gene delivery systems [84,85]. Among various graphene-based materials, graphene oxide (GO) is a flexible layered material with large specific surface area and aromatic sp^2^ domains [86,87]. On its basal surface and edges, there are many functional groups such as hydroxyl, epoxy, carboxyl, and carbonyl, which make it easy to disperse in water and physiological environments [88]. As a result of its large specific surface area, GO can be used to load or immobilize various drugs and biological molecules by π–π stacking and hydrogen bond interactions. GO also has pH-responsive drug release behavior, where enhanced drug releases at a low pH value, which is suitable for biomedical application. Therefore, GO combined with Fe_3_O_4_ NPs can be beneficial in improving their properties for targeted drug/gene delivery systems [33,34,35].

Magnetic graphene oxide (mGO) was synthesized by chemical co-precipitation of Fe_3_O_4_ NPs on GO nanoplatelets [63]. Then mGO was modified with chitosan through covalent binding to synthesize chitosan-modified mGO (mGOC). mGOC was subsequently grafted with PEG to form PEGylated mGOC (mGOC-PEG), which avoids endocytosis by the RES and prolongs blood circulation of the nanocarrier. Irinotecan (CPT-1) or DOX was loaded with mGOC–PEG through π–π stacking and hydrogen bond interactions for magnetic targeted chemotherapeutic drug delivery. With high drug loading, pH-dependent drug-controlled release properties, and magnetic targeting, mGOC–PEG would be a promising drug carrier to enhance the efficacy of cancer therapy in vivo. Similarly, Song et al. also prepared GO via a modified Hummers’ method, and Fe_3_O_4_ NPs were loaded on the GO surface through a chemical precipitation method to form Fe_3_O_4_@GO nanocomposites [35]. Then, lactoferrin (Lf) was linked with Fe_3_O_4_ NPs by the amide linkage between amino groups on Lf and the carboxyl groups of PEG-coated Fe_3_O_4_ NPs, which could be bonded on the surface of receptors overexpressing glioma cells, and as a targeted ligand to further improve the targeting properties of the nanocomposites. Finally, DOX was loaded onto Lf-linked Fe_3_O_4_@GO nanocomposites by π–π stacking to form Lf@GO@Fe_3_O_4_@DOX nanocomposites. Lf@GO@Fe_3_O_4_@DOX nanocomposites exhibited better intracellular delivery efficiency and higher cytotoxicity against C6 glioma cells in comparison with free DOX and DOX@GO@Fe_3_O_4_ nanocomposites.

**Figure 3:**
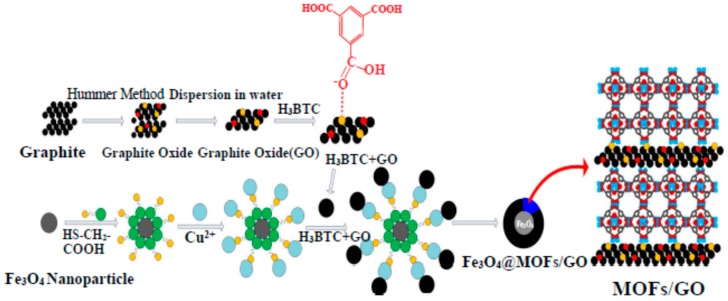
Illustration of the fabrication of Fe_3_O_4_@MOFs/GO microspheres [79]. MOFs: metal-organic frameworks; GO: graphene oxide.

By exploring the layer-by-layer self-assembly method and the changes of content of GO and the repeat time of the reaction procedure, controllable synthesis of a core-shell microsphere Fe_3_O_4_@MOFs/GO (MOFs: metal-organic frameworks) with different layers and content of GO was achieved by mixing with 1,3,5-benzenetricarboxylic acid (H_3_BTC) (Figure 3) [79]. The results show that the drug-loading capacity of 20-layer core-shell spheres for ibuprofen was better than 10 layers with the same GO content, implying that the drug-loading capacity could be optimized by tuning the shell thickness. Meanwhile, the drug-loading capacity was enhanced with increasing GO content. However, when the content of GO was increased to 15%, GO aggregation was induced by hydrogen bonding, and then the adsorption capacity of GO was decreased and the drug-loading efficiency was reduced. These results show that GO plays a decisive role in using Fe_3_O_4_@MOFs/GO in drug-delivery systems. The core-shell microsphere with 10% GO content was the suitable candidate for drug-delivery systems in this work. These results show that microspheres with 20 layers had better drug-releasing capacity than those with 10 layers. Deng et al. also used a simple and feasible layer-by-layer technique to fabricate a hybrid microcapsule (h-MC) consisting of polysaccharides, Fe_3_O_4_ NPs, and GO [33]. The linking of multilayer polysaccharide coatings comprising sodium alginate (Alg), chitosan (Chi), and hyaluronic acid (HA) to obtain the microcapsule with Fe_3_O_4_@GO was denoted as Alg/Chi/Fe_3_O_4_@GO/Chi/HA (h-MC), and the microcapsule without Fe_3_O_4_@GO was denoted as Alg/Chi/Alg/Chi/HA (MC). DOX was used as a drug model to load on the polysaccharide layers. Compared to MC, the on-demand drug liberated from h-MC could be realized under a magnetic field or mild near-infrared light irradiation, and the controllability of drug release was greatly improved. In addition, the polysaccharides ensured greater bioavailability and tumor accumulation. Hence, a micro-matryoshka for on-demand drug liberation and hyperthermia was harvested.

### 3.4. Carbon Coating

Other carbon coating materials are also widely applied for the protection and stabilization of Fe_3_O_4_ NPs due to their unique properties, such as superior chemical and thermal stability, biocompatibility, and fluorescent imaging ability. In addition, there are numerous functional groups on the surface of carbon coating that enable it to be easily modified and functionalized, and to be utilized in many areas of biomedicine.

Zhao et al. developed the synthesis of Fe_3_O_4_/C core/shell structures with different shell thicknesses under hydrothermal conditions [53]. Ammonium acetate (CH_3_COONa) was applied as a structure-guiding agent, which promotes the concentrated growth of carbon coating on the surface of Fe_3_O_4_ NPs and inhibits the development of giant pure carbon microspheres. A simple, low-energy, and environmentally friendly method for the preparation of advanced carbon-coated material is proposed in this study, and other coating materials could be employed to further function on the microspheres for many applications. A novel Fe_3_O_4_/C magnetic composite was also fabricated by another research group via an effortless in-suit one-pot template-free solvothermal reaction in the presence of ion (III) precursor and mesoporous carbon with the same carbon source, sucrose, for the loading of ciprofloxacin antibiotic [37]. Multicolor fluorescent carbon dot-attached Fe_3_O_4_ NPs through the use of meta-phenylenediamine and para-phenylenediamine as carbon sources, respectively, were reported by Pramanik et al. [38]. The formed nanocomposites could be used for antibody conjugation, and MRI-fluorescent dual imaging was also realized, which exhibited excellent potential for use in clinical settings. Wang et al. also used a novel precursor material, ferrocene, to directly fabricate carboxyl-modified Fe_3_O_4_@C nanospheres [78]. The organic linkers and surface-adsorbed metal ions were bonded on the surface of the Fe_3_O_4_@C nanospheres via a layer-by-layer approach to form Fe_3_O_4_@C@MIL-100(Fe) (FCM) nanoparticles. The small carbon dots could be embedded in the middle carbon layer of the obtained FCM nanoparticles, which were employed as two-photon fluorescence imaging contrast agents, and the Fe^3+^ and drug could be loaded on the outer MIL-100(Fe) shell. Therefore, the formed FCM nanoparticles could be utilized for MRI and two-photon fluorescence dual-modal imaging and targeted delivery of anticancer drug and Fe^3+^.

Metal-organic frameworks (MOFs), series of hybrid materials consisting of metal ions and organic bridging ligands, have attracted much attention due to their tunable properties and ability to be utilized for separation [89], heterogeneous catalysis [90], sensing [91], gas storage [92], and cargo delivery [93]. Recently, MOFs have been used as a fluorescent detection sensor for DNA and proteins [94]. Tan et al. used iron-containing MOFs (MIL-88A; MIL: Materials Institute Lavoisier) as precursors via the one-pot thermolysis method to synthesize a porous carbon-coated magnetic Fe_3_O_4_ NP nanocomposite and investigated its application to fluorescent sensing for DNA detection [95].

### 3.5. Other Coating Materials

The carbonyl iron powders, carbon nanotubes, and gelatin microcapsules can be employed as microwave-absorbing materials to improve the effectiveness of microwave treatment [96,97]. However, these materials generate extra heat in tumor tissues and their toxicity, biocompatibility, and biodegradability in vivo need to be further evaluated. These shortcomings have largely limited their utilization in the biomedical field, particularly in vivo. Metal oxide microwave-absorbing materials such as Fe_3_O_4_, ZnO, TiO_2_, ZrO_2_, and WO_3_ have superior microwave absorbing capacity, unique responsiveness to microwave heat, chemical stability, and low toxicity, which make them a better candidate for microwave thermo-seed host materials [74,98].

Peng et al. have demonstrated fabrication of core-shell-structured Fe_3_O_4_@mZnO nanocomposites via a simple, environmentally friendly, and template-free homogeneous precipitation approach [75]. The surface area (93 m^2^·g^−1^) and magnetization saturation value (40.3 emu·g^−1^) of the as-prepared nanocomposites were relatively higher, which made them exploitable for drug loading and targeting. The as-prepared nanocomposites also had excellent drug-loading ability and the microwave was converted to thermal after microwave irradiation, which could provide a platform for a combination of chemotherapy and microwave thermotherapy in vivo. Qiu et al. also used ZnO as a microwave-absorbing material to fabricate Fe_3_O_4_@ZnO@mSiO_2_ nanocarrier, and the ZnO nanoparticles were synthesized by a precipitation process with zinc acetate [39]. Compared with 53% of the etoposide (VP16) loading in the above-mentioned Fe_3_O_4_@mZnO nanocomposites, the drug loading could increase to approximately 70% due to the presence of a mesoporous silica shell. Under intermittent microwave irradiation, the release amount of VP16 could go up to more than 85%, but only 14% of the drug was released within 10 h of stirring. Hence, the as-prepared multifunctional system offered a superior candidate for microwave-triggered targeted drug delivery.

In general, microwave absorption capacity improves with reduced forbidden bandwidth. The forbidden bandwidth of WO_3_ (2.7 eV) is far less than those of ZnO (3.37 eV) and TiO_2_ (3.2 eV), which gives WO_3_ nanomaterials superior microwave thermal response performance [99]. However, it is very hard for them to form a core-shell structure, which hinders their application. Due to magnetite having a sensitive quenching impurity for rare earth luminescent materials, if lanthanide-doped nanoparticles are utilized as cores of the multifunctional nanocomposites and Fe_3_O_4_ nanoparticles as shells, the upconversion luminescence emission intensity of lanthanide is reduced, due to the absorption of excitation and emission light by magnetic shells. While lanthanide-doped nanoparticles are directly coated on the surface of Fe_3_O_4_ nanoparticles, the optical quenching is caused by energy transfer and charge transfer. The preparation of lanthanide-doped shell layers always needs high-temperature treatment, and the magnetic materials are destroyed, leading to a reduction of magnetic property, which also limits the formation of a core-shell structure. Therefore, an inorganic inert interlayer, such as SiO_2_, is needed. The SiO_2_ interlayer not only avoids optical quenching and decreased magnetization, but also protects the magnetic nanoparticles from agglomeration. Zhao et al. employed WO_3_ as the microwave thermal translation material to fabricate an (3-aminopropyl) trimethoxysilane (APTES)-modified magnetic core-shell-targeted drug-delivery system (Fe_3_O_4_@WO_3_@mSiO_2_-APTES) [76]. The WO_3_ and mSiO_2_ nanoparticles enabled the nanocomposite to have a relatively larger surface area of 234.5 m^2^·g^−1^ for drug loading, a higher magnetization saturation value of 40.54 emu·g^−1^ for targeted drug delivery via external magnetic fields, and a higher reflection loss of −22.75 dB for the controlled release of drug under microwave irradiation. The release of VP16 was more than 85.69% with the use of microwave discontinuous irradiation, while the drug release was 15.88% with 600 min of stirring. Thus, compared with ZnO or TiO_2_ as a microwave-absorbing agent, the nanocomposite that contained WO_3_ had better characteristics for drug load and release carriers.

The lanthanide-doped nanoparticles combine with Fe_3_O_4_ NPs to form multifunctional nanocomposites, which have applications in biomedicine, such as MRI, optical probes, cell separation, labeling, and imaging, and controlled administration of sustained-release drugs [100,101]. If lanthanide-doped nanoparticles are utilized as cores of multifunctional nanocomposites, the luminescent intensity of the nanocomposites will be repressed to a certain extent, caused by the presence of the outer layers [102], while lanthanide-doped nanoparticles are directly coated on the surface of Fe_3_O_4_ NPs, then induce an emergence of fluorescence quenching, which causes their luminescent ability to be reduced [103]. Thus, it is necessary to contact an interlayer, such as silica, between Fe_3_O_4_ NPs and lanthanide-doped nanoparticles. The silica interlayer not only protects and modifies the Fe_3_O_4_ NPs and limits the agglomeration or corrosion of particles, but also provides some bonding sites for further modification with lanthanide-doped nanoparticles.

**Figure 4:**
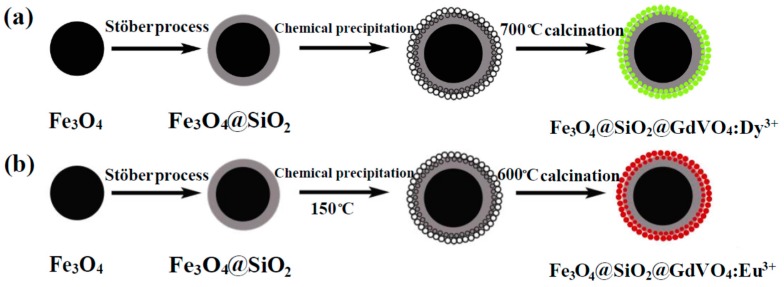
Illustration of the preparation process of (**a**) Fe_3_O_4_@SiO_2_@GdVO_4_:Dy^3+^ and (**b**) Fe_3_O_4_@SiO_2_@GdVO_4_:Eu^3+^ nanocomposites [55,104].

Li et al. developed a simple method for the fabrication of Fe_3_O_4_@SiO_2_@GdVO_4_:Dy^3+^ core-shell-structured nanocomposites, as shown in Figure 4a [55]. The results of a cytotoxicity analysis showed that when the concentration of nanocomposites increased to the maximal value of 200 μg·mL^−1^ for 24 h, HeLa cell viability remained at 90.38%, proving that the nanocomposites were biocompatible. Meanwhile, the high saturation magnetization and magnetic response of the Fe_3_O_4_@SiO_2_ nanoparticles, and the GdVO_4_:Dy^3+^ with excellent fluorescence properties and high specific surface area, ensured that the nanocomposites could be applied in MRI, drug delivery, and cell labeling and separation. Later, this group also prepared Fe_3_O_4_@SiO_2_@GdVO_4_:Eu^3+^ multifunctional nanocomposites with a similar method (Figure 4b) [104]. The cytotoxicity analysis of the nanocomposites suggested that cell viability was still at 89.38%, even at the maximum concentration of 200 μg·mL^−1^. This demonstrated that this kind of nanocomposite had superior biocompatibility and could also be utilized for targeted drug delivery with MRI-fluorescence imaging.

## 4. Fe_3_O_4_ NPs for Targeted Drug/Gene Delivery Systems

### 4.1. Fe_3_O_4_ NP Drug Delivery of Chemotherapeutic Agents

Chemotherapy is one of the most extensively applied techniques for antitumor treatment. However, it has many shortcomings, such as systemic toxicity [105], lower treatment efficacy, and multidrug resistance, which hamper its clinical use. Therefore, structuring of targeted drug-delivery systems with superior therapeutic effects and minimal toxicity is urgently needed. Fe_3_O_4_ NPs have received great interest due to their unique magnetic performance, excessively low cytotoxicity, excellent biocompatibility and biodegradability, and various reactive sites for contact with drugs after being modified with various coatings. The carried drugs accumulate in cancer cell sites under the influence of an external magnetic field through the enhanced permeability and retention effect [106,107]. Thus, the poisonous side effects of a drug will be minimized, with improved treatment efficacy. Meanwhile, considering the characteristics of the coatings, the combination of chemophotothermal therapy, targeted drug delivery, MRI, and other therapeutic methods will be achieved [28,30,31,45,59]. Recently, various drugs loaded with functionalized Fe_3_O_4_ NPs have been reported, and in the following sections we introduce several common drug-loaded delivery systems [25,26,72,73]. After being modified with diverse coating materials, the loaded drugs will be released based on different triggers, such as common pH-responsive, NIR laser-triggered, temperature-sensitive, shell-thickness-dependent, ROS-mediated cytotoxicity, microwave-triggered, interaction with DNA to produce a therapeutic effect, and low-frequency ultrasound. Different cell lines (in vivo or in vitro) were used for analysis of the toxicity and treatment efficacy of some modified nanoparticles, as shown in Table 1.

#### 4.1.1. Doxorubicin

Doxorubicin (DOX), an anthracycline antibiotic, has been used for tumor therapy, because it can prevent the synthesis and transcription of DNA via the interaction between DNA nucleotides and the inhibition of topoisomeraseII. As with other chemotherapy drugs, destructive radicals are also generated during the metabolism of DOX and act on the proliferating cells, then inhibition of DNA synthesis and the division and metabolism of cells are disturbed. However, healthy cells are also damaged in this process, which causes poisonous side effects. Thus, combining DOX with Fe_3_O_4_ NPs for targeted drug delivery will be indispensable.

**Figure 5:**
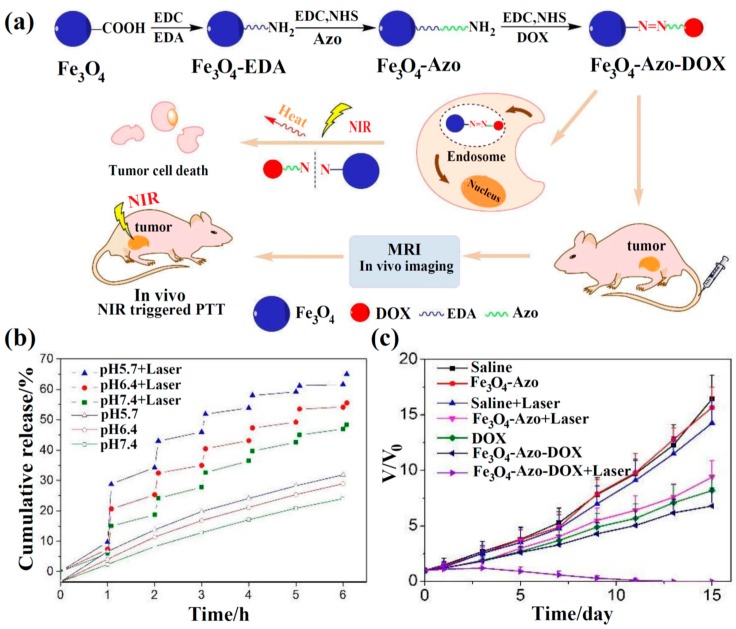
(**a**) Schematic illustration of the synthesis of a thermosensitive drug-delivery system based on Fe_3_O_4_–Azo NPs for chemo-photothermal therapy in vitro and in vivo; (**b**) DOX release from Fe_3_O_4_–Azo NPs at pH 5.7, pH 6.4, and pH 7.4 with and without irradiation (The laser groups were irradiated repeatedly over a period of 5 min, followed by 1 h intervals without irradiation.); (**c**) Relative growth curves of tumors in different treatment groups within 15 days [77].

Zhang et al. reported a modified polyol method to fabricate PEG and PEI coatings of Fe_3_O_4_ NPs, and then folic acid (FA) was functionalized and DOX as a drug model was loaded on the nanoparticles to form DOX@FA–Fe_3_O_4_ NPs [49]. Wu et al. fabricated 4,4-azobis (4-cyanovaleric acid) (Azo)-functionalized multifunctional Fe_3_O_4_ NPs (Fe_3_O_4_–Azo NPs), which allowed NIR laser-dependent drug release and photothermal chemotherapy (Figure 5a,b) [77]. First, 1,2-ethanediamine was attached to the surface of Fe_3_O_4_ NPs. By utilizing Azo as a pyrolysis coupling agent, DOX connected with the nanoparticles via covalent bond to obtain Fe_3_O_4_–Azo–DOX NPs. Under NIR irradiation, the Fe_3_O_4_ NPs absorbed NIR light and quickly converted to thermal with the increased temperature. When it reached the critical fracture temperature of 43 °C, the Azo was cleaved and released DOX in the tumor-targeted sites, and tumor growth was inhibited. The combined chemo-photothermal therapy results are shown in Figure 5c. There was no noticeable growth inhibition of cancer cells in saline, Fe_3_O_4_–Azo NPs, and saline + laser groups. The cancer cells treated under free DOX, Fe_3_O_4_–Azo NP + laser, and Fe_3_O_4_–Azo–DOX NP groups could be inhibited to a certain extent, but the tumor volume still enlarged by 8.2, 9.4, and 6.8 times, respectively. Compared with other treatment groups, when the Fe_3_O_4_–Azo–DOX NP + laser group was used to incubate in mice, the growth of cancer cells was speedily suppressed and completely eliminated after 13 days, and no tumor recidivism was found. These results show that in comparison to chemotherapy or photothermal therapy alone, combined chemo-photothermal therapy with the use of Fe_3_O_4_–Azo–DOX NPs and laser displayed more powerful growth inhibition of tumor, and there was no remarkable toxicity to major organs such as liver, heart, lung, spleen, and kidney. In summary, the as-prepared Fe_3_O_4_–Azo–DOX NPs had an influential anticancer effect and lower general acute toxicity, enabling them to offer a promising platform for targeted drug-delivery systems and combined chemotherapy and photothermal therapy.

Su et al. discovered a new graphene quantum dot (GQD)@Fe_3_O_4_@SiO_2_ nanoprobe, which could be used for not only targeted drug delivery, but also MRI and fluorescent dual imaging and therapy systems [108]. Then, a targeting molecule, folic acid (FA), was attached to this nanoprobe and loaded with DOX drug molecules to obtain Fe_3_O_4_@SiO_2_@GQD–FA/DOX nanoprobes. These four components were combined into a fluorescence resonance energy transfer system. Once DOX was loaded onto the GQD surface via π–π stacking interaction, the fluorescence emission of GQD was quenched through fluorescence resonance energy transfer. Thus, at this assembly stage, the fluorescence of GQD was at “off” status, while DOX fluorescence was at “on” status. Subsequently, the nanoprobes were internalized by targeted tumor cells and the DOX was released under an acidic environment accompanied by turning the GQDs to “on” status. The results demonstrate that turning on the GQD/DOX fluorescence signals permitted the tracking of nanoprobes and the release process of the drug. The prepared Fe_3_O_4_@SiO_2_, Fe_3_O_4_@SiO_2_@GQD, and Fe_3_O_4_@SiO_2_@GQD–FA all exhibited superior biocompatibility and cell viability greater than 80%, and the Fe_3_O_4_@SiO_2_@GQD–FA nanocarrier possessed excellent cell viability even at a concentration up to 100 μg·mL^−1^. The cells treated with Fe_3_O_4_@SiO_2_@GQD/DOX and Fe_3_O_4_@SiO_2_@GQD–FA/DOX had greater inhibition of tumor compared to free DOX at the initial concentration, which indicated that by using the nanoprobes, therapeutic efficacy was greatly improved. In addition, when the concentration of DOX was 10 μg·mL^−1^, HeLa cells presented lower viability in Fe_3_O_4_@SiO_2_@GQD–FA/DOX (15.4%), in contrast to Fe_3_O_4_@SiO_2_@GQD/DOX (20.4%), which could be ascribed to the targeting ligand FA further improving the targeting efficacy of carriers. These results demonstrate that the as-formed Fe_3_O_4_@SiO_2_@GQD–FA/DOX versatile nanoprobe with distinctive magnetic, fluorescence, and targeting properties offers a new way of thinking about the combination of targeted tumor diagnosis, therapy, and drug-molecule tracking.

From the above examples, we can see that pH/NIR laser irradiation-triggered drug release, fluorescence imaging tracking of drug load and release, and enhanced targeting and combined therapy can be accomplished by applying DOX-loaded multifunction-modified Fe_3_O_4_ NPs, and then DOX can enable more optimal treatment for cancer patients.

#### 4.1.2. 5-Fluorouracil

5-Fluorouracil (5-FU) is the oldest pyrimidine analogue of antimetabolite, and is widely employed as an antineoplastic drug for numerous solid cancers, including tumors of the intestine, pancreas, breast, stomach, colon, ovaries, and other cancers, alone or in combination. However, the half-life of 5-FU in vivo is shorter, which causes a lower drug concentration in blood and a reduced treatment effect [109]. Thus, to boost treatment efficacy, larger doses of the drug or continual medication will be chosen for patients, resulting in poisonous side effects of 5-FU. To overcome these problems, various 5-FU-loaded nanoparticles have been developed for targeted medicine sustained-release systems [64,110].

Prabha et al. described the synthesis and characterization of 5-FU-loaded Fe_3_O_4_–CD, Fe_3_O_4_–CD–PEG, and Fe_3_O_4_–CD–PEG–PEI nanocomposites as promising targeted drug carriers [65]. Their research demonstrated that similar to the above-mentioned Fe_3_O_4_–CS–PEG–PVP nanocomposites, with an increased number of composite coatings, the electrostatic attraction and hydrogen bond interactions between these nanocomposites and the drugs increased, and the EE and LC of nanocomposites were enhanced at similar 5-FU concentrations. This phenomenon indicates that Fe_3_O_4_–CD–PEG–PEI was the most favorable drug carrier in this study. The drug release times of these nanocomposites increased after coating with polymers, and a slow release of drugs was achieved, due to the high polymeric chain, increased hydrophilic properties, and reduced absorption via RES, all leading to water entering slowly. At pH 6.8, the release rate of drug was improved due to the larger degrees of nanocomposites, and the increased temperature also promoted the release of drugs, so that targeted drug release would be obtained by the application of a foreign magnetic field. Subsequently, the cytoactivity of mouse fibroblast cells (L929) and breast cancer cells (MCF-7) in free 5-FU, Fe_3_O_4_–CD–5-FU, Fe_3_O_4_–CD–5-FU–PEG, Fe_3_O_4_–CD–5-FU–PEG–PEI, and unloaded Fe_3_O_4_–CD–PEG–PEI was explored. This study indicated that all of these nanocomposites were biocompatible with L929 cells, which was proved by the activity of 80% of cells. In MCF-7 cell groups, only the unloaded Fe_3_O_4_–CD–PEG–PEI was nontoxic, while the other nanocomposites were cytotoxic, and with an increase of the initial 5-FU concentration, the cytotoxicity of Fe_3_O_4_–CD–5-FU–PEG–PEI was significantly higher than that of Fe_3_O_4_–CD–5-FU or Fe_3_O_4_–CD–5-FU–PEG. All of these experiments confirmed that the Fe_3_O_4_–CD–PEG–PEI nanocomposites were the most suitable carriers for targeted drug delivery systems in this study.

Likewise, Aliabadi et al. described a 5-FU–loaded magnetic nanocomposite, which was combined with Fe_3_O_4_ NPs, GO, chitosan, and polyvinyl alcohol (PVA) [66]. The LC and EE of this nanocomposite were 35.9% and 88.9%, respectively, indicating that it could be utilized as a nanocarrier in vivo. It also had pH-responsive drug-release properties; the drug release amount of this nanocomposite reached 91.9% under an acidic environment (pH 5.8), which was higher than at pH 7.4 (80.6%). This could be attributed to the presence of chitosan, which could increase under acidic conditions because the amine groups were protonated and the increased polymer chains caused the larger repulsion. Meanwhile, the existing PVA in the composite could improve the circulation time of drugs in blood and reach targeted tissue in a given time period. These superior properties enable this nanocomposite to be better used for targeted drug delivery in cancer treatment.

#### 4.1.3. Ciprofloxacin

Ciprofloxacin is a third-generation synthetic fluoroquinolone antibiotic that has broad-spectrum antimicrobial activity; bacteria are killed by inhibition of DNA synthesis and replication [111]. The antibacterial activity is the most efficient compared to other fluoroquinolone antibiotics such as norfloxacin and enoxacin. Thus, it is extensively utilized for the treatment of urinary tract and intestinal infections. However, the short half-life of about 4–5 h leads to larger doses or more frequent administration, which limits its clinical utilization, and controlled-release systems are urgently needed.

Shamsipur et al. described the fabrication of chitosan (CS)-modified Fe_3_O_4_ NPs, and then ciprofloxacin as a drug model was loaded on the surface of the nanoparticles by hydrogen bond and/or electrostatic interactions [71]. The loading of ciprofloxacin of nearly 99% was noticeably high, which allowed for reduced dosing and counteracted the side effects and toxic effects, thereby optimizing the therapeutic effect. Without ultrasound, the drug released relatively continuously for 400 min, and then gradually over the next five days. On the other hand, 95% of the drug released extremely rapidly from the nanoparticles within 60 min under low-frequency ultrasound. This phenomenon could be interpreted as the effect of ultrasound, one of the most significant foreign triggers for controlled drug release [112], loosening the tight skeleton of polymer chains and allowing the water and drug to pass through the polymer easily, causing an effective release of the drug. Based on the points discussed above, a system for targeted and controlled release of ciprofloxacin could be achieved by exposing drug-loaded CS-coated Fe_3_O_4_ NPs to an external magnetic field and ultrasonic triggers.

#### 4.1.4. Gemcitabine

Gemcitabine (Gem) is a nucleoside analogue antimetabolic anticarcinoma agent that can incorporate with DNA so that the nucleotides cannot be connected with DNA polymerase, causing the cessation of chain elongation and, further, apoptosis of cancer cells. It has been researched in various tumors such as pancreatic, non-small-cell lung, breast, ovarian, and bladder cancer [113]. However, it has a short half-life of about 8–17 min, resulting in the need for larger doses of the drug to achieve the required plasma concentrations, hence creating serious systemic toxicity and side effects, drug resistance, and lower therapeutic efficacy. Besides this, it must enter the cells by nucleoside transporters due to its hydrophilic properties, and cannot be transported into the cells via passive transportation [114]. Thus, a higher nucleoside transporter expression in patients is required to obtain a considerably higher survival rate, which restricts the use of Gem in patients with lower levels of nucleoside transporters. To overcome the above two shortcomings, Parsian et al. synthesized CS-coated Fe_3_O_4_ NPs and loaded Gem on the nanoparticles [69]. The cytotoxicity assays showed that CS-coated Fe_3_O_4_ NPs were not significantly toxic to SKBR and MCF-7 cells when the concentration was up to 0.5 mg·mL^−1^. Gem-CS-coated Fe_3_O_4_ NPs increased the cellular uptake of Gem by endocytosis and caused more cytotoxicity, and their inhibitory concentration (IC_50_) values were nearly 2.6- and 1.4-fold lower in comparison to free Gem on SKBR and MCF-7 cells, respectively. Therefore, the treatment efficacy of Gem was promoted by use of the nanoparticles.

#### 4.1.5. Dihydroartemisinin

**Figure 6:**
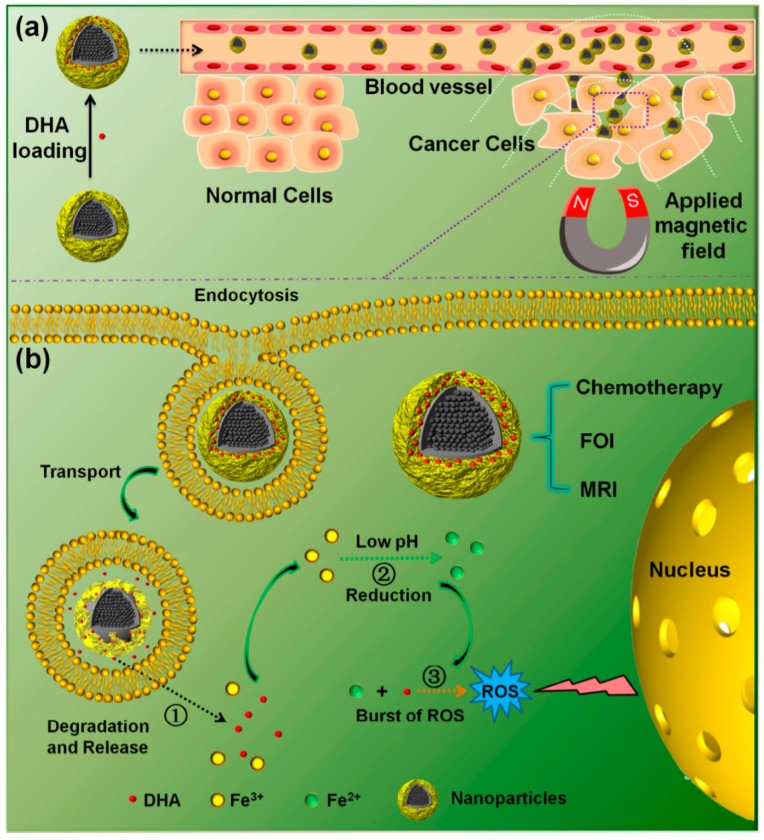
(**a**) Schematic illustration of targeting of dihydroartemisinin (DHA)-loaded Fe_3_O_4_@C@MIL-100(Fe) (FCM) nanoparticles to tumor cells assisted by an externally applied magnetic field; and (**b**) the anticancer mechanism of the DHA delivery system [78].

Artemisinin, a sesquiterpene lactone drug in the peroxide group, is extracted from the leaves of the Artemisia annua plant and used for the treatment of malaria. Its semisynthetic derivative dihydroartemisinin (DHA) is able to interact with Fe^2+^ to generate reactive oxygen species (ROS) causing cytotoxicity [115], potentially reducing or eliminating the multidrug resistance of traditional chemotherapy agents. However, the lack of Fe^3+^ and synchronous delivery of DHA and Fe^3+^ in cancer cells make this therapeutic method a dilemma. Wang et al. reported that Fe_3_O_4_@C@MIL-100(Fe) (FCM) nanoparticles for dual-model imaging-targeted drug-delivery systems had pH-dependent and magnetic targeted drug-release behaviors [78]. In the nanoparticles, the outer MIL-100(Fe) shells are capable of co-carrying DHA and Fe^3+^ to avoid this dilemma. When the nanoparticles were exposed in the acidic environments of inflammation and cancer cells, degradation of MIL-100(Fe) shells occurred, with a synchronous release of DHA and Fe^3+^. Then, the pH of the endosome was lowered, and there was reduction of Fe^3+^ to Fe^2+^ through ferric reductase and other molecules with reducibility of the cell. Finally, Fe^2+^ reacted with the simultaneously released DHA to produce a certain amount of cytotoxic ROS, then lipids were oxidized and damaged membranes, proteins, and nucleic acid, causing the death of cancer cells (Figure 6). In in vivo animal models, significant uptake of cancer cells by nanoparticles was achieved under a magnetic field, and a complete cure of cancer cells was observed with no obvious toxicity or side effects in healthy tissues or animal behavior. Based on the above experiments, the novel fabricated nanoparticles could be used as a succedaneum for the conventional clinical model in the cure of cancer.

The efficient generation of toxic ROS also plays an important role in treatment effects of other anticancer drugs, such as platinum drugs, including cisplatin, oxaliplatin, and carboplatin, DOX, and artesunate. Another PEI, PEG, and rhodamine B modified Fe_3_O_4_ nanocarrier was also constructed to facilitate ROS production of cisplatin, DOX, and artesunate [73]. In this nanocarrier, drug induced the production of H_2_O_2_, and Fe_3_O_4_ NPs degraded and were metabolized in cancerous cells, with the release of iron ions so that H_2_O_2_ was decomposed catalytically into highly toxic hydroxyl radicals and caused fast oxidation and deterioration of cancerous membranes. It can be concluded that under the auxiliary function of Fe_3_O_4_ nanocarrier, the toxic ROS intensity dramatically increased and the IC_50_ of such drugs would decrease, eventually producing an improved antitumor effect. In short, this Fe_3_O_4_ nanocomposite could be an outstanding carrier for such ROS-mediated cytotoxicity drugs.

### 4.2. Fe_3_O_4_ NP Gene Delivery for Gene Therapy

Gene therapy is inseparable from gene carriers, because when individual genes are injected into the body, they will be rapidly degraded and damaged by humoral substance and enzymes, and the negatively charged macromolecule nucleic acids are hampered in approaching and passing through the cell membrane with the same charge due to electrostatic repulsion. At the same time, with the rigorous research of Fe_3_O_4_ NPs and the persistent exploration of its applications, we found that Fe_3_O_4_ NPs could be used not only to deliver chemotherapy drugs, but also to transfer genes to tumor sites. Thus, targeted gene transfer systems will be engineered [36,47,116]. Moreover, gene delivery systems are able to prevent nucleic acids from enzymatic degradation and boost the cellular internalization and targeted release of the genes, improving the treatment effect.

When employing Fe_3_O_4_ NPs as gene transfers, they are usually coated with organic ligands, such as PEG, PEI, aminosilanes, chitosan, or polyamidoamine (PAMAM) dendrimer [32,117]. The gene would be linked on the carrier typically through electrostatic interactions between gene and polymer; gold coating and lipid membrane could also support anchorage points for genes to be connected. Then targeted gene carriers consisting of Fe_3_O_4_ nanocomposites would be obtained. Some gene-loaded Fe_3_O_4_ nanocomposites carriers are shown in Table 2. The PEI/DNA complex is usually used as a nonviral gold standard for other gene carriers. However, once the PEI/DNA complex is employed in a simulated physiological environment or in vivo, serum protein interference will be caused by PEI and its derivatives, resulting in decreased transfection efficiency. Meanwhile, PEI also has non-targeting properties, potential toxicity, and host immunogenic effects in vivo. These disadvantages all limit its application in targeted gene delivery. Combining Fe_3_O_4_ NPs with PEI will allow the high magnetofection to address these limitations. Zhang et al. displayed Fe_3_O_4_ NP magnetofection carrier for enhanced transfection of red fluorescent protein encoding plasmid DNA, which applied polymer modules comprising chitosan and PEG copolymer (CP) modified with catechol (CCP). CCP was coated on the surface of Fe_3_O_4_ NPs (IOCCP), and PEI was further modified with CCP (IOCCP-PEI) for the binding of DNA [32]. Significantly, DNA-loaded IOCCP-PEI had outstanding transfection effect in vitro, and further functionalization with PEG (IOCCP-PEI-PEG) greatly decreased cytotoxicity without obviously hampering the transfection effect. So, the optimal IOCCP-PEI-PEG gene carrier could also be utilized for the binding of other DNA in a targeted gene delivery system. A magnetic Fe_3_O_4_ NP PEI/DNA complex gene carrier (MPD-cc) for enhanced green fluorescent protein encoding plasmid DNA was also prepared by mixing carboxylic acid-silane-modified magnetic Fe_3_O_4_ NPs and PEI/DNA complexes [117]. Compared to traditional PEI/DNA (PD), less serum protein was absorbed on MPD-cc during magnetofection. After 4 h of treatment, cells in PD and MPD-cc without magnetic field groups exhibited fragmentary green fluorescent spots, while the fluorescent intensity was highly enhanced after magnetofection under MPD-cc for 10 min. Other PEI-modified superparamagnetic iron oxide nanocomplexes (PSPIOs) were also developed for effective magnetofection of the same DNA and MRI of human cancer cells [47]. Similarly, in contrast with ordinary PEI/DNA complexes, the PSPIO complexes not only showed better colloidal stability in a serum-containing environment, superior targeting, lower cytotoxicity, and more effective transfection, but also rendered T_2_ contrast agents for MRI in cancer cells. From the above analysis, we can see that combining Fe_3_O_4_ NPs with tradition PEI/DNA complexes under a foreign magnetic field would allow high magnetofection and MRI in cancer cells, breaking new ground for the synthesis of targeted gene delivery systems.

Another coating for PAMAM dendrimers, a kind of spherical polymer with a larger number of end groups on the surface, can be attached with plasmid DNA and oligonucleotides to obtain stable complexes. Gunduz et al. described a three-layer dendrimer-coated magnetic nanoparticle (DcMNP) consisting of an Fe_3_O_4_ NP magnetic core, an aminosilane interlayer, and PAMAM dendrimers for the targeted delivery of CpG-oligodeoxynucleotides (CpG-ODNs) [118]. CpG-ODNs can interact with and activate Toll-like receptor 9 (TLR9) [119], stimulating the immune system to produce a sequence of signals about cell death. There are characteristic unmethylated CpG-ODN sequences in bacterial and viral DNA. TLR9 expression is generated not only in most cells of the immune system, but also in tumor cells, such as brain, lung, gastric, prostate, and breast cancer [120,121]. Unmethylated CpG-ODNs of vertebrates can be specifically recognized by TLR9 and located at endoplasmic reticulum, then transfer to the endosomal or lysosomal compartment to identify and link with ligand. Subsequently, cell internalization signaling mediators are produced by TLR9 and its related aptamers, thereby activating transcription factors. The activated TLR9 induces apoptosis of various pernicious cells, including breast tumor cells. Therefore, the TLR9 analeptic including CpG-ODNs could open new possibilities for curing tumors. Additionally, exploiting the CpG-loaded DcMNP could extend the circulation life of the nanoparticles, and the CpG-ODNs could easily access the cancer cells and concentrate on the target sites, reducing the amount of CpG-ODNs added, thus the IC_50_ of CpG-ODNs for breast cancer cells would be reduced and effective treatment could be achieved. DcMNP also supplies an efficient and suitable nanocarrier to transfer other therapeutic nucleotides to cancer cells in biomedical uses.

Tumor-targeting components such as antibodies, aptamers, cell-penetrating peptides, and small molecules such as folic acid are usually exploited to link with Fe_3_O_4_ NPs, which further enhances the targeting of the Fe_3_O_4_ NPs in gene delivery. Among them, antibodies are the most specific target components, as their production processes are detailed and complicated and they have potential immunogenicity, also causing the size of Fe_3_O_4_ NPs to enlarge, leading to a reduction of their cellular internalization, all of which restricts their exploitation in the modification of Fe_3_O_4_ NPs [122]. On the other hand, the easily accessible cell-penetrating peptides and small molecules are commonly nonspecific to particular cancer cells. For instance, folic acid, mentioned in the above drug/gene delivery systems, is only specific to folate receptor overexpressing cancerous cells and not specific to various types of cancerous cells. Consequently, aptamers are received as the desirable targeted reagents. Aptamers are short oligonucleotide sequences via in vitro screening with high affinity and specificity to the corresponding ligands; for example, the distinction of subtypes of non-small-cell lung cancer could be actualized by highly specific aptamers [123]. Meanwhile, various aptamers could be grafted on the surface of Fe_3_O_4_ NPs to obtain multiple binding and synergistic affinity. Dinarvand et al. engineered gold coating-modified Fe_3_O_4_ NPs (Au@Fe_3_O_4_ NPs), then grafted thiol-modified oligonucleotide MUC-1 aptamer as a targeting reagent to synthesize aptamer-Au@Fe_3_O_4_ NPs, which actualized drug-free MRI and photothermal therapy of colon cancer [124]. After the HT-29 human colon cancer cell line and Chinese hamster ovarian cell line (CHO) were treated with Au@Fe_3_O_4_ NPs and aptamer-Au@Fe_3_O_4_ NPs, the cellular uptake of nanoparticles was confirmed by confocal microscopy. Compared with exposure to Au@Fe_3_O_4_ NPs, the HT-29 cells in aptamer-Au@Fe_3_O_4_ NPs had greater fluorescence intensity, indicating that these cells absorbed more targeted nanoparticles. However, there was the same intake of these two nanoparticles in CHO cells, confirming the targeting of aptamer-Au@Fe_3_O_4_ NPs. Results of the photothermal therapeutic experiments demonstrated that cancerous cells were completely eliminated at 200 μg·mL^−1^ and 500 μg·mL^−1^ with radiation of NIR light, and at the most effective 500 μg·mL^−1^ of aptamer-Au@Fe_3_O_4_ NPs, 80% of tumor cells were killed. With radiation of NIR light, the Au@Fe_3_O_4_ NPs had less effect on cells. The photothermal properties of Au@Fe_3_O_4_ NPs and the low toxicity of the laser were proven, and the heat resistance of normal cells was better than that of tumor cells, so photothermal therapy based on Au@Fe_3_O_4_ NPs is a feasible and safe approach for cancer. However, the exact mechanism of cell death after photothermal therapy is still unclear and many possibilities, such as plasma membrane disruption, ROS-mediated apoptosis, and DNA damage, all need further study.

### 4.3. Fe_3_O_4_ NP Drug/Gene Co-Delivery for Chemo–Gene Combined Therapy

In general, the use of drug or gene therapy alone usually fails to actualize the desired curative effects. Therefore, it is imperative to structure a single co-delivery system for the combination of chemotherapy drugs and gene therapy. In this co-delivery system, the combined action of drug and gene on cancerous cells will be realized, and then not only will tumors be cured from the disease origin by gene correction and replacement, but also the drug will cause apoptosis and death of tumor cells. A co-delivery system has the following advantages over a conventional drug or gene delivery system alone: it reduces injection time for treatment, improves compliance and the patient’s quality of life, and there is a synergistic or superimposed treatment effect at lower dosage, thus toxic side effects will be decreased and better curative effects will be achieved. In addition, the interaction between certain genes and specific drugs can enhance the therapeutic effects, including drugs promoting the expression of therapeutic genes, and the sensitivity of tumor cells to drugs will be elevated by the genes and the tolerance and resistance of cancerous cells to drugs will be reduced. Various co-delivery systems have been designed for chemotherapeutic agents and nucleic acid drugs; among them, Fe_3_O_4_ NPs are the most developed system with hyperthermal therapy, MRI, and in vivo tracking and other advantages [125,126,127].

Li et al. engineered and constructed magnetic Fe_3_O_4_@mSiO_2_ NPs (M-MSNs) and then modified them with PEI and FA for co-delivery of plasmid-expressing small hairpin RNA against vascular endothelial growth factor (VEGF shRNA) and DOX (M-MSN(DOX)/PEI-FA/VEGF shRNA) [128]. In vitro cytotoxicity assays confirmed that the M-MSN and M-MSN/PEI-FA nanocomplexes had no obvious toxicity on HeLa cells or HUVECs (normal cells) and had superior biocompatibility. Without a magnetic field, the cell viability of HeLa cells treated with M-MSN(DOX)/PEI-FA/VEGF shRNA nanocomplexes was lower than the that of cells incubated with M-MSN(DOX)/PEI-FA and M-MSN/PEI-FA/VEGF shRNA nanocomplexes alone, which made clear that the penetration and uptake of DOX in tumor cells were increased by VEGF shRNA and a synergistic or superimposed treatment effect could be obtained. Under an external magnetic field, the cells in M-MSN(DOX)/PEI-FA/VEGF shRNA nanocomplexes had the lowest cell viability, only 27%, and the therapeutic efficacy of DOX was further facilitated. The silencing effect of VEGF expression was assessed to eliminate interference from DOX-caused apoptosis. M-MSN/PEI-FA/VEGF shRNA displayed a higher targeted gene-silencing effect of 90.4% by FA-mediated cellular uptake and effectively inhibited the immigration of endothelial cells and the formation of capillary-like structures caused by transfected HeLa cells, resulting in effective treatment. In summary, an excellent targeted co-delivery system of drug and gene for tumor therapy was engineered, which offers an optimal treatment effect and a new perspective for combination gene and drug therapy for tumor cells.

**Figure 7:**
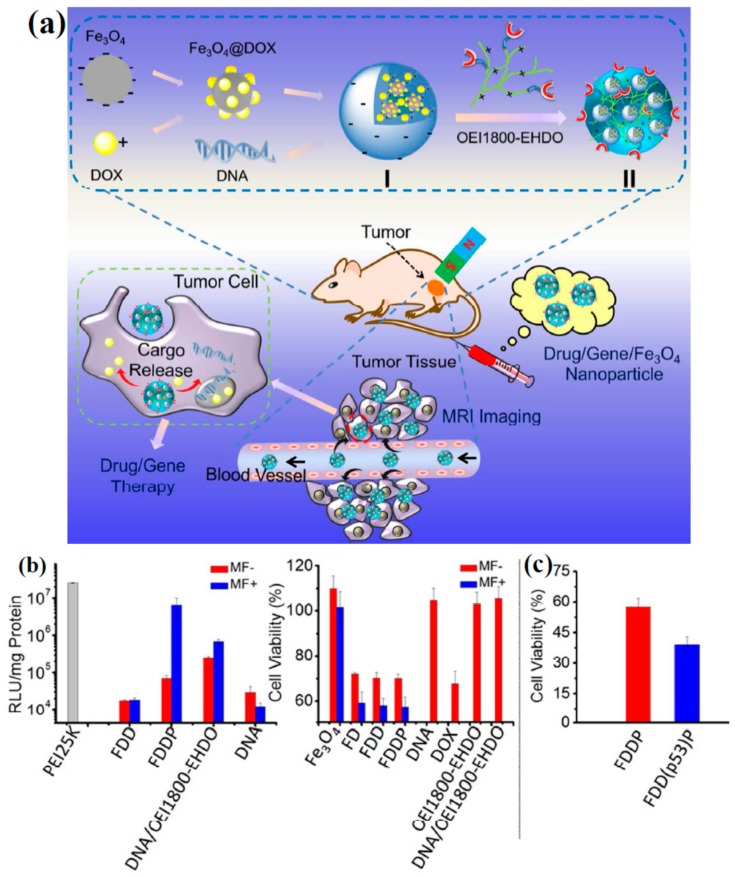
(**a**) Synthesis process of the Fe_3_O_4_@DOX/DNA/OEI1800-EHDO (FDDP) nanosystem for magnetic targeted drug/gene co-delivery and MRI; (**b**) transfection activity and cell cytotoxicity of various samples in HeLa cells after 30 min of treatment with/without magnetic field; and (**c**) cytotoxicity of DOX and pGL-3 co-loaded FDDP NPs and DOX and p53 co-loaded FDD(p53)P NPs in HeLa cells under magnetic field [129].

The all-in-one nanoparticles that could entrap Fe_3_O_4_ NPs, DOX and DNA for the magnetic targeting, MRI, the delivery of gene and drug and the chemo-gene combined therapy had also been established as shown in Figure 7a [129]. The following OEI1800-EHDO (5-ethyl-5-hydroxymethyl-1,3-dioxan-2-oxo modified polyethylenimine with the molecular weight of 1800 Da) was mixed with FDD NPs to obtain Fe_3_O_4_@DOX/pGL-3/OEI1800-EHDO (FDDP) NPs, which was owing to the inductive polycations OEI1800-EHDO could be utilized for the construction of nanoplatformand, and prevent DNA against the premature degradation and provide effective uptake via negatively charged cells, based on its convenient noncatalyst synthesis, excellent biocompatibility, higher plasma tolerance and easy to expand [130]. Then the feasibility of the nanosystems as co-delivery carriers was evaluated. The DOX entrapment efficiency could be as high as 70%, which was suitable for the drug delivery. Furthermore, this nanosystem vastly improved the chances of gene and drug entering cells under magnetic field and the ability of cell internalization. The satisfactory co-delivery systems not only possess the skill of loading, transferring and liberating the drugs and genes to the targeting sites also keeping their individual properties at top levels. Consequently, the drug release efficacy and gene transfection of the nanosystems were discussed with or without magnetic field trigger. The transfection rate of OEI1800-EHDO/pGL-3 and the cytotoxicity of the concurrent release DOX interfered with each other at minimal level, and the toxicity of loaded DOX could contend with free drug at the commensurable dose. Following the substitution of non-treatment effect pGL-3 with p53 gene possess apoptosis induction to confirm the anticancer effective of the chemo-gene combined therapy in this nanosystem, and the formed nanosystem was marked as FDD(p53)P. Under magnetic field, compared to the cell viability of 60% under FDDP NPs, the viability of cells incubated with FDD(p53)P was further reduced to 35.7% (Figure 7b,c), which exhibited that the synergistic treatments were resulting. These above results implied that the all-in-one nanosystem capacitated the effective co-delivery of loaded cargoes to targeted cells and the obtainment of superlative combination therapeutic effects, and could boost the proceeding of co-delivery nanosystems for other different versatile employments.

## 5. Conclusions and Future Prospects

Currently, there is great interest in Fe_3_O_4_ NPs in biomedicine due to their outstanding performance. In this review, we first pay close attention to the fabrication of Fe_3_O_4_ NPs and outline the three commonly used methods: co-precipitation and solvothermal and thermal decomposition. Because the as-fabricated naked Fe_3_O_4_ NPs have inherited defects of ready aggregation and instability, functionalization with numerous coatings is imperative. The resulting functionalized Fe_3_O_4_ NPs not only contain a magnetic core with excellent magnetism for MRI and magnetic force-triggered targeting locations, but the linked coatings are hypotoxic, biocompatible, and biodegradable, and also have various function groups for the conjugation of therapeutic agents and further modification, and their excellent performance can be applied to other therapies, such as thermal treatment, fluorescent imaging probes, and pH-/temperature-/time-triggered or microwave-activated drug/gene delivery, for effective combined therapy. Thus, functionalized Fe_3_O_4_ NPs open up a brand-new horizon for the targeted delivery of drugs or genes in cancer therapies. The loaded drugs have high solubility in fluids, superior pharmacokinetics, and selective biodistribution in illness sites, providing extraordinarily effective treatment and minimal systemic toxicity in tumor chemotherapy. Similarly, the linked genes are easily internalized by the targeted cells, accompanied by a reduction of degrading and destruction by enzymes or other humoral substances. Thus, gene transfection is significantly promoted with the boosting of a gene-silencing effect, improving the effect of gene therapy. Unfortunately, drug or gene monotherapy nanosystems are usually hampered by a lack of the desired curative effects. As a result, we describe Fe_3_O_4_ NP co-delivery systems that can accommodate drugs and genes and have magnetic targeting-induced transfer and release to provide maximum synergistic effects for drug and gene combined therapy, so that an unimpeded path can be opened up for the treatment of cancer patients.

However, in other respects, functionalized Fe_3_O_4_ NP targeted delivery systems still face multiple difficulties and challenges for practical use in clinical transformation. First, the methods of functionalizing Fe_3_O_4_ nanoparticles include ligand addition and ligand exchange. Ligand addition is limited by increased hydrodynamic diameter, and for ligand exchange, the size of nanoparticles is not magnified, but the ratio of nanoparticles to ligands is uncertain and the process is time-consuming and labor-intensive, and the efficiency of ligand exchange is lower with longer time duration. Hence, we must work on how to obtain efficient ligand exchange during the functionalizing procedure of converting water-insoluble Fe_3_O_4_ nanoparticles to hydrophilic Fe_3_O_4_ NPs. Commercial production of Fe_3_O_4_ NPs and drug-/gene-loaded functionalized Fe_3_O_4_ NPs at a large scale, rather than just at the magnitude of several hundred milligrams, will be indispensable for clinical transformation. Second, as is known, there are intrinsic differences between animal models and persons suffering from cancer, thus considering the security of clinical employment, some problems are intractable. It is essential to fully investigate the features of as-formed targeted delivery systems involving biocompatibility, biodegradation, biodistribution, and low cytotoxicity and stability, and on-demand targeting release in the human body. Sustained work is needed to explore the mechanism by which as-prepared Fe_3_O_4_ NPs enter human diseased cells and how they interact with human cancerous cells, as well as the mechanism of cell death and the pathways of cell/body metabolism. We firmly believe that these barriers will be overcome by our unremitting efforts. There can be little doubt, however, that Fe_3_O_4_ NP-based targeted drug/gene delivery systems are new, extraordinarily valuable methods that will play a pivotal role in the field of biomedicine and open up a novel area of research.

## Figures and Tables

**Table 1:** Functionalization of Fe_3_O_4_ NPs with different coatings for targeted drug delivery.

Cell Lines	Loaded Drugs	Coating Materials	Releasing Factors		Refs.
**Co-precipitation**	Doxorubicin (DOX)	Carboxymethyl chitosan (CS)	pH−	MCF-7, S180 (in vivo/in vitro)	[28]
	DOX	Sodium alyinate (SA), chitosan (CS), graphene oxide (GO), hyaluronic acid (HA)	pH−, near-infrared (NIR)	HeLa (in vivo/in vitro)	[33]
	DOX	Lactoferrin, GO	pH−	C6 (in vitro)	[35]
	DOX	Poly(*N*-isopropylacrylamide) (PNIPAAM), 3-(trimethoxysilyl) propyl methacrylate (TMSPMC)	pH−, thermosensitive	–	[62]
	DOX, irinotecan	CS, GO, methoxypolyethylene glycol succinimidyl carbonate ester (mPEG-NHS)	pH−	U87 (in vitro)	[63]
	5-Fluorouracil (5-Fu)	Zr(HPO_4_)_2_·H_2_O, folic acid (FA), CS, R6G	pH−	A549, HEK293, HeLa (in vitro)	[64]
	5-Fu	β-cyclodextrin, polyethylenimine (PEI), polyethyline glycol (PEG),	Shell thickness, pH−, temperature	L929, MCF-7 (in vitro)	[65]
	5-Fu	GO, CS, polyvinyl alcohol (PVA)	pH−	–	[66]
	Curcumin	FA, polyamidoamine (PAMAM)	FA receptor	SKOV3, HeLa (in vitro)	[25]
	Curcumin	Silk fibroin	Silk fibroin concentration, pH−	MDA-MB-231 (in vitro)	[61]
	Curcumin	CS, PEG, polyvinylpyrrolidone (PVP)	Shell thickness, pH−	Caco-2, HCT-116 (in vitro)	[67]
	C6	FA, GO, Oleic acid (OA)	Light- and reductive-triggered	HeLa, A549 (in vitro)	[34]
	C6, e6	OA, silane	Light irradiation photodynamic	MCF-7 (in vivo/in vitro)	[59]
	Methoterxate (MTX)	Gold layer, Lipoic acid-PEG	NIR	KB, MRC-5, 4T1 (in vivo/in vitro)	[26]
	Nimustine, semustine, chlormethine	CA	Interact with DNA or prevent DNA relaxation	MHCC97-H, MCF-7 (in vitro)	[16]
	nicotinamide	SiO_2_	DNA binding interaction	-	[17]
	Cytarabine	SiO_2_	DNA binding interaction	HL-60, KG-1, Raji (in vitro)	[68]
	*N*-[*N*-(3,5-Difluor-ophenacetyl-l-al-anyl)]-*S*-phenylg-lycinet-butylester (DAPT)	Polypyrrole (PPy), HA	pH−	4T, MDA-MB-231, MCF-7 (in vitro)	[31]
	Gemcitabine	CS	pH−	SKBR, MCF-7 (in vitro)	[69]
	Heteropolyacids (HPAs)	Starch-*g*-poly(EP) hydrogel biopolymer	Hydrolysis of polymer chains	–	[70]
	Ciprofloxacin	CS	Low-frequency ultrasound	–	[71]
**Thermal Decomposition**	DOX	Gold nanorods and nanoclusters, bovine serum albumin (BSA)	NIR, magnetic triggered	HeLa (in vitro)	[46]
	DOX	Graphene quantum dot, SiO_2_, FA	pH−, fluorescence resonance energy transfer (FRET)	HeLa (in vitro)	[51]
	DOX	PEG, PEI, FA	pH−	MCF-7 (in vivo/in vitro)	[49]
	Cisplatin	PEI, Gd_2_O_3_, FA, PEG	pH−, reactive oxygen species (ROS)-mediated toxicity	HeLa, NHLF (in vivo/in vitro)	[72]
	Cisplatin, DOX, artesunate	PEG, PEI, rhodamine B	pH−, ROS-mediated toxicity	A2780, ACP (in vivo/in vitro)	[73]
	Mycophenolic acid (MPA)	SiO_2_	Release MPA by hydrolysis in cells	Peripheral blood mononuclear cells (PBMCs) (in vitro)	[48]
	VP16	ZnO, mSiO_2_	Microwave-triggered, pH−, temperature	–	[39]
	VP16	ZnO, Gd_2_O_3_:Eu, P(NIPAm-*co*-MAA)	Microwave, pH−	–	[74]
	VP16	mZnO	Microwave	–	[75]
	VP16	WO_3_, mSiO_2_, (3-aminopropyl) trimethoxysilane (APTES)	Microwave, pH−, temperature	–	[76]
**Solvothermal Synthesis**	DOX	Azo	pH−, NIR	MCF-7, S180 (in vivo/in vitro)	[77]
	5-Fu	PNIPAAM, mSiO_2_, CS, R6G	Thermoresponsive drug release	7901 (in vitro)	[30]
	Dihydroartemisinin	C and MIL-100 (Fe)	pH, ROS-mediated cytotoxicity	A549, HeLa (in vivo/in vitro)	[78]
	HSP70	Polydopamine	NIR	HCT116 (in vitro)	[29]
	Ibuprofen	Metal-organic frameworks, GO	Drug release controlled by layers	–	[79]

**Table 2:** Functionalization of Fe_3_O_4_ NPs with different coatings for targeted gene delivery.

Preparation Method	Coating Materials	Loaded Gene	Gene Connection	Cell Lines	Refs.
**Co-precipitation**	CS, PEG, catechol, PEI	pRFP DNA	Electrostatic interactions between PEI and plasmid DNA	SF767 human glioblastoma multiforme (GBM) (in vitro)	[32]
	Chlorotoxin, CS, PEG, PEI	Green fluorescent protein (GFP) encoding DNA	Electrostatic interactions between PEI and DNA	C6 (in vivo)	[80]
	CA-silane, PEI	p-encoding green fluorescent protein (pEGFP), pGL3, pCMV-Luc	Electrostatic interactions of PEI with DNA and carboxylic acid	HepG2 (in vitro/in vivo)	[117]
	PAMAM dendrimer	CpG oligodeoxynucleotide	Electrostatic interactions between PAMAM and DNA	MDA-MB231, SKBR3 (in vitro)	[118]
**Thermal Decomposition**	Dopamine, PEG-NH_2_	DNA, Pcambia, PGEM-T	Electrostatic interactions between amino groups and plasmid DNA	Escherichia coli cells (in vitro)	[19]
	PEG, liposomes, gold	Chol-DNA	Au coating provided anchorage points for DNA to be attached	–	[60]
	PEI	DNA constructed by pGL3-basic to pcDNA3 vector	Electrostatic interactions between PEI and plasmid DNA	ALTS1C1, PC3, HEK293T (in vitro)	[47]
**Solvothermal Synthesis**	Ethanolamin-functionalized poly(glycidyl methacrylate), SiO_2_, APTES	EGFP encoding plasmid DNA	Electrostatic interactions between linked polymer and plasmid DNA	HepG2, C6, HEK293 (in vitro/in vivo)	[36]
